# Supplementary material for: AffyMAPSDetector: a software tool to characterize Affymetrix GeneChip™ expression arrays with respect to SNPs
Source: BMC Bioinformatics. 2007 Jul 30;8:276. doi: 10.1186/1471-2105-8-276 (PMC1959249; doi:10.1186/1471-2105-8-276)
Supplement: Additional file 13 — AffyMAPSDetector v1 source code. AffyMAPSDetector source code. [file 1471-2105-8-276-S13.zip › AffyMAPSDetector_v1_src/doc/jwf/NullWizardPanel.html]

NullWizardPanel


|  |  |  |  |
| --- | --- | --- | --- |
| **Overview** \| **Package** \| **Class** \| **Use** \| **Tree** \| **Deprecated** \| **Index** \| **Help** | | |  |
| **PREV CLASS**   **NEXT CLASS** | **FRAMES**    **NO FRAMES**     **All Classes** |  |  |
| SUMMARY: NESTED \| FIELD \| CONSTR \| METHOD | DETAIL: FIELD \| CONSTR \| METHOD |  |  |


---


## jwf Class NullWizardPanel

```
java.lang.Object
  java.awt.Component
      java.awt.Container
          javax.swing.JComponent
              javax.swing.JPanel
                  jwf.WizardPanel
                      jwf.NullWizardPanel
```

**All Implemented Interfaces:**: javax.accessibility.Accessible, java.awt.image.ImageObserver, java.awt.MenuContainer, java.io.Serializable

---

public class **NullWizardPanel** extends WizardPanel

An implementation of the base class used for implementing a panel that is
displayed in a Wizard. Used if a null panel is set.

**Author:**
:   Christopher Brind

**See Also:**: Serialized Form

---

**Nested Class Summary**

**Nested classes inherited from class javax.swing.JPanel**

`javax.swing.JPanel.AccessibleJPanel`

**Nested classes inherited from class javax.swing.JComponent**

`javax.swing.JComponent.AccessibleJComponent`

**Nested classes inherited from class java.awt.Container**

`java.awt.Container.AccessibleAWTContainer`

**Nested classes inherited from class java.awt.Component**

`java.awt.Component.AccessibleAWTComponent, java.awt.Component.BltBufferStrategy, java.awt.Component.FlipBufferStrategy`


**Field Summary**

**Fields inherited from class jwf.WizardPanel**

`wizardContext`

**Fields inherited from class javax.swing.JComponent**

`accessibleContext, listenerList, TOOL_TIP_TEXT_KEY, ui, UNDEFINED_CONDITION, WHEN_ANCESTOR_OF_FOCUSED_COMPONENT, WHEN_FOCUSED, WHEN_IN_FOCUSED_WINDOW`

**Fields inherited from class java.awt.Component**

`BOTTOM_ALIGNMENT, CENTER_ALIGNMENT, LEFT_ALIGNMENT, RIGHT_ALIGNMENT, TOP_ALIGNMENT`

**Fields inherited from interface java.awt.image.ImageObserver**

`ABORT, ALLBITS, ERROR, FRAMEBITS, HEIGHT, PROPERTIES, SOMEBITS, WIDTH`


**Constructor Summary**

`NullWizardPanel()`
  
          A default constructor.


|  |  |
| --- | --- |
| **Method Summary** | |
| `boolean` | `canFinish()`             Can this panel finish the wizard? |
| `void` | `display()`             Called when the panel is set. |
| `void` | `finish()`             Handle finishing the wizard. |
| `boolean` | `hasNext()`             Is there be a next panel? |
| `WizardPanel` | `next()`             Get the next panel to go to. |
| `boolean` | `validateFinish(java.util.List list)`             Called to validate the panel before finishing the wizard. |
| `boolean` | `validateNext(java.util.List list)`             Called to validate the panel before moving to next panel. |

**Methods inherited from class jwf.WizardPanel**

`getWizardContext, hasHelp, help, setWizardContext`

**Methods inherited from class javax.swing.JPanel**

`getAccessibleContext, getUI, getUIClassID, paramString, setUI, updateUI`

**Methods inherited from class javax.swing.JComponent**

`addAncestorListener, addNotify, addPropertyChangeListener, addPropertyChangeListener, addVetoableChangeListener, computeVisibleRect, contains, createToolTip, disable, enable, firePropertyChange, firePropertyChange, firePropertyChange, firePropertyChange, firePropertyChange, firePropertyChange, firePropertyChange, firePropertyChange, firePropertyChange, fireVetoableChange, getActionForKeyStroke, getActionMap, getAlignmentX, getAlignmentY, getAncestorListeners, getAutoscrolls, getBorder, getBounds, getClientProperty, getComponentGraphics, getConditionForKeyStroke, getDebugGraphicsOptions, getDefaultLocale, getGraphics, getHeight, getInputMap, getInputMap, getInputVerifier, getInsets, getInsets, getListeners, getLocation, getMaximumSize, getMinimumSize, getNextFocusableComponent, getPreferredSize, getPropertyChangeListeners, getPropertyChangeListeners, getRegisteredKeyStrokes, getRootPane, getSize, getToolTipLocation, getToolTipText, getToolTipText, getTopLevelAncestor, getTransferHandler, getVerifyInputWhenFocusTarget, getVetoableChangeListeners, getVisibleRect, getWidth, getX, getY, grabFocus, isDoubleBuffered, isLightweightComponent, isManagingFocus, isMaximumSizeSet, isMinimumSizeSet, isOpaque, isOptimizedDrawingEnabled, isPaintingTile, isPreferredSizeSet, isRequestFocusEnabled, isValidateRoot, paint, paintBorder, paintChildren, paintComponent, paintImmediately, paintImmediately, print, printAll, printBorder, printChildren, printComponent, processComponentKeyEvent, processKeyBinding, processKeyEvent, processMouseMotionEvent, putClientProperty, registerKeyboardAction, registerKeyboardAction, removeAncestorListener, removeNotify, removePropertyChangeListener, removePropertyChangeListener, removeVetoableChangeListener, repaint, repaint, requestDefaultFocus, requestFocus, requestFocus, requestFocusInWindow, requestFocusInWindow, resetKeyboardActions, reshape, revalidate, scrollRectToVisible, setActionMap, setAlignmentX, setAlignmentY, setAutoscrolls, setBackground, setBorder, setDebugGraphicsOptions, setDefaultLocale, setDoubleBuffered, setEnabled, setFont, setForeground, setInputMap, setInputVerifier, setMaximumSize, setMinimumSize, setNextFocusableComponent, setOpaque, setPreferredSize, setRequestFocusEnabled, setToolTipText, setTransferHandler, setUI, setVerifyInputWhenFocusTarget, setVisible, unregisterKeyboardAction, update`

**Methods inherited from class java.awt.Container**

`add, add, add, add, add, addContainerListener, addImpl, applyComponentOrientation, areFocusTraversalKeysSet, countComponents, deliverEvent, doLayout, findComponentAt, findComponentAt, getComponent, getComponentAt, getComponentAt, getComponentCount, getComponents, getContainerListeners, getFocusTraversalKeys, getFocusTraversalPolicy, getLayout, insets, invalidate, isAncestorOf, isFocusCycleRoot, isFocusCycleRoot, isFocusTraversalPolicySet, layout, list, list, locate, minimumSize, paintComponents, preferredSize, printComponents, processContainerEvent, processEvent, remove, remove, removeAll, removeContainerListener, setFocusCycleRoot, setFocusTraversalKeys, setFocusTraversalPolicy, setLayout, transferFocusBackward, transferFocusDownCycle, validate, validateTree`

**Methods inherited from class java.awt.Component**

`action, add, addComponentListener, addFocusListener, addHierarchyBoundsListener, addHierarchyListener, addInputMethodListener, addKeyListener, addMouseListener, addMouseMotionListener, addMouseWheelListener, bounds, checkImage, checkImage, coalesceEvents, contains, createImage, createImage, createVolatileImage, createVolatileImage, disableEvents, dispatchEvent, enable, enableEvents, enableInputMethods, getBackground, getBounds, getColorModel, getComponentListeners, getComponentOrientation, getCursor, getDropTarget, getFocusCycleRootAncestor, getFocusListeners, getFocusTraversalKeysEnabled, getFont, getFontMetrics, getForeground, getGraphicsConfiguration, getHierarchyBoundsListeners, getHierarchyListeners, getIgnoreRepaint, getInputContext, getInputMethodListeners, getInputMethodRequests, getKeyListeners, getLocale, getLocation, getLocationOnScreen, getMouseListeners, getMouseMotionListeners, getMouseWheelListeners, getName, getParent, getPeer, getSize, getToolkit, getTreeLock, gotFocus, handleEvent, hasFocus, hide, imageUpdate, inside, isBackgroundSet, isCursorSet, isDisplayable, isEnabled, isFocusable, isFocusOwner, isFocusTraversable, isFontSet, isForegroundSet, isLightweight, isShowing, isValid, isVisible, keyDown, keyUp, list, list, list, location, lostFocus, mouseDown, mouseDrag, mouseEnter, mouseExit, mouseMove, mouseUp, move, nextFocus, paintAll, postEvent, prepareImage, prepareImage, processComponentEvent, processFocusEvent, processHierarchyBoundsEvent, processHierarchyEvent, processInputMethodEvent, processMouseEvent, processMouseWheelEvent, remove, removeComponentListener, removeFocusListener, removeHierarchyBoundsListener, removeHierarchyListener, removeInputMethodListener, removeKeyListener, removeMouseListener, removeMouseMotionListener, removeMouseWheelListener, repaint, repaint, repaint, resize, resize, setBounds, setBounds, setComponentOrientation, setCursor, setDropTarget, setFocusable, setFocusTraversalKeysEnabled, setIgnoreRepaint, setLocale, setLocation, setLocation, setName, setSize, setSize, show, show, size, toString, transferFocus, transferFocusUpCycle`

**Methods inherited from class java.lang.Object**

`clone, equals, finalize, getClass, hashCode, notify, notifyAll, wait, wait, wait`

**Constructor Detail**

### NullWizardPanel

```
public NullWizardPanel()
```

:   A default constructor.


**Method Detail**

### display

```
public void display()
```

:   Called when the panel is set.

    :   **Specified by:**: `display` in class `WizardPanel`

---


### hasNext

```
public boolean hasNext()
```

:   Is there be a next panel?

    :   **Specified by:**: `hasNext` in class `WizardPanel`
    :   **Returns:**: true if there is a panel to move to next

---


### validateNext

```
public boolean validateNext(java.util.List list)
```

:   Called to validate the panel before moving to next panel.

    :   **Specified by:**: `validateNext` in class `WizardPanel`
    :   **Parameters:**: `list` - a List of error messages to be displayed. **Returns:**: true if the panel is valid,

---


### next

```
public WizardPanel next()
```

:   Get the next panel to go to.

    :   **Specified by:**: `next` in class `WizardPanel`

---


### canFinish

```
public boolean canFinish()
```

:   Can this panel finish the wizard?

    :   **Specified by:**: `canFinish` in class `WizardPanel`
    :   **Returns:**: true if this panel can finish the wizard.

---


### validateFinish

```
public boolean validateFinish(java.util.List list)
```

:   Called to validate the panel before finishing the wizard. Should
    return false if canFinish returns false.

    :   **Specified by:**: `validateFinish` in class `WizardPanel`
    :   **Parameters:**: `list` - a List of error messages to be displayed. **Returns:**: true if it is valid for this wizard to finish.

---


### finish

```
public void finish()
```

:   Handle finishing the wizard.

    :   **Specified by:**: `finish` in class `WizardPanel`


---


|  |  |  |  |
| --- | --- | --- | --- |
| **Overview** \| **Package** \| **Class** \| **Use** \| **Tree** \| **Deprecated** \| **Index** \| **Help** | | |  |
| **PREV CLASS**   **NEXT CLASS** | **FRAMES**    **NO FRAMES**     **All Classes** |  |  |
| SUMMARY: NESTED \| FIELD \| CONSTR \| METHOD | DETAIL: FIELD \| CONSTR \| METHOD |  |  |


---
